# Which antithrombotic strategy provides the best outcomes after mitral valve repair in patients who remain in sinus rhythm?

**DOI:** 10.1093/icvts/ivac085

**Published:** 2022-04-19

**Authors:** Jason Trevis, Enoch Akowuah

**Affiliations:** 1 Academic Cardiovascular Unit, South Tees NHS Foundation Trust, Middlesbrough, TS4 3BW, UK; 2 Newcastle University, Framlington Place, Newcastle-upon-Tyne, NE2 4HH, UK

**Keywords:** Mitral valve repair, Anticoagulation, Antithrombotic, Antiplatelet, Sinus rhythm

## Abstract

A best evidence topic in cardiac surgery was written according to a structured protocol. The question addressed was ‘in the first 3-months after mitral valve repair (MVRep) which antiplatelet and/or anticoagulant strategy should be instigated in patients who remain in normal sinus rhythm’. Altogether 77 papers were found using the reported search, of which 8 represented the best evidence to answer the clinical question. The authors, journal, date and country of publication, patient group studied, study type, relevant outcomes and results of these papers are tabulated. We conclude that there remains a lack of high-quality randomized studies, controlling for postoperative cardiac rhythm, comparing vitamin K antagonists (VKA) and antiplatelet therapy in the early postoperative period following isolated MVRep. Current guidelines are based on limited evidence or expert consensus alone. Based on the currently available evidence, the authors conclude that antiplatelet therapy (e.g. aspirin) is safe and appropriate to use in the 3-month postoperative period following isolated MVRep, in those without preoperative, or postoperative atrial fibrillation. Rates of thromboembolic events are comparable between these patient groups (i.e. VKA versus aspirin), whilst VKA therapy is associated with increased rates of major bleeding events and mortality.

## INTRODUCTION

A best evidence topic was constructed according to a structured protocol. This is fully described in the *ICVTS* [1].

## THREE-PART QUESTION

In patients undergoing [mitral valve repair] which [anticoagulation and/or antiplatelet strategy] provides [the best outcomes in the first three post-operative months].

## CLINICAL SCENARIO

A 70-year-old patient, with a history of hypertension, is consented for mitral valve repair (MVRep) due to a degenerative mitral regurgitation. The procedure is uneventful, and the patient remains in sinus rhythm throughout the admission. You know that there is a particularly increased risk of thromboembolic events in the first 3 months following MVRep due to ongoing endothelialization of the prosthetic material. The patient has no background of AF preoperatively, however you recognize the increased incidence of postoperative new-onset AF within this patient group. You liaise with your colleagues within the department, who give varying strategies for thromboembolic prophylaxis in this patient group. Consequently, you search for what the best short-term postoperative antiplatelet/anticoagulant strategy would be for this patient according to the best evidence.

## SEARCH STRATEGY

Searched EMBASE using the OVID interface, utilizing the following search strategy: [mitral valve repair/OR mitral valve surgery/] AND [antithromb$.mp. OR anticoagulation/OR anticoagulant agent/OR anticoag$.mp. OR antiplatelet.mp] AND [outcome assessment/OR survival/OR complication/OR morbidity/OR mortality/OR stroke.mp OR cerebrovascular accident/OR thromboemol$.mp.]

## SEARCH OUTCOME

A total of 77 papers were found using the reported search. From these, 8 papers were identified that provided the best evidence to answer the question. These are presented in Table 1.

**Table 1:** Best evidence articles

Author, date and country Study type (level of evidence)	Patient group	Outcomes	Key results	Comments
Whitlock *et al.* (2012), Chest, Canada [3] Guideline (level 1a)	Guideline on antithrombotic and thrombolytic therapy for valvular disease, from the ACCP	Recommendation for patients undergoing mitral valve repair	Antiplatelet therapy for the first 3 months over VKA therapy. No randomized trial to evaluate the use of antithrombotic therapy after mitral valve repair	Recommendation based upon observational data; recognizing the limitations in controlling the study population with regard to prevalence of atrial fibrillation
Vahanian *et al.* (2012), Eur Heart J, France [4] Guideline (level 1a)	Guideline on the management of valvular heart disease, from the ESC and EACTS Joint Task Force	Recommendation for patients undergoing mitral valve repair	‘Oral anticoagulation should be considered for the first 3 months after mitral valve repair’	
Sousa-Uva *et al.* (2017), Eur J Cardiothorac Surg, Portugal [5] Guideline (level 1a)	Guideline on perioperative medication in adult cardiac surgery, from the EACTS Task Force	Recommendation for patients undergoing mitral valve repair	Oral anticoagulation with VKA for the first 3 months	Risk of thromboembolic and bleeding complication should be accounted for
Dunning *et al.* (2008), Eur J Cardiothoracic Surg, UK [6] Guideline (level 1a)	Guideline on antiplatelet and anticoagulation management in cardiac surgery, from the EACTS Audit and Guidelines Committee	Recommendation for patients undergoing mitral valve repair	‘Patients who have an indication (e.g. atrial fibrillation) should be anticoagulated’ ‘Antiplatelet therapy alone is an acceptable alternative’	Anticoagulation for others may be beneficial and is reasonably safe
Nishimura *et al.* (2014), Circulation, USA [7] Guideline (level 1a)	Guideline on the management of patients with valvular heart disease, from the AHA and ACC	Recommendation for patients undergoing mitral valve repair	Anticoagulation with a VKA for the first 3 months after bioprosthetic mitral valve replacement or repair	To achieve an INR of 2.5. After 3 months VKA can be discontinued, unless the patient has associated risk factors, e.g. atrial fibrillation, previous thromboembolism or hypercoagulable condition
Paparella *et al.* (2016), J Thorac Cardiovasc Surg, Italy [8] Retrospective cohort study (level 2b)	Study period: 2011–2013 Propensity matched sample; *n* = 1144 VKA group: *n* = 858 Antiplatelet group (APLT) (100 mg aspirin daily): *n* = 286	6 months postoperative Primary efficacy outcome: Incidence of arterial thromboembolic event Primary safety outcome: Incidence of major bleeding 6-Month mortality	Propensity-matched analysis: APLT versus VKA 2.1% vs 1.6% *P* = 0.5 0.7% vs 3.9% *P* = 0.01 0.3% vs 2.7% *P* = 0.02	Data on those who developed atrial fibrillation following discharge was not recorded Primary safety outcome data measured up to 6 months following repair or the stop of VKA + 1 day, depending on which came first
van der Wall *et al.* (2018), J Thromb Thrombolysis, The Netherlands [9] Retrospective cohort study (level 2b)	Study period: 2004–2016 Sample: *n* = 469 VKA group = 325 Aspirin group (80 mg daily): *n* = 144	3 months postoperative Primary end point: Combined incidence of thromboembolic and major bleeding complications (those who remained in sinus rhythm) Secondary end point(s): Incidence of thromboembolic events Incidence of major bleeding events	VKA versus aspirin 8.2% vs 8.1% Adjusted HR 0.97, 95% CI 0.32–2.9 2.6% vs 1.6% Adjusted HR 0.82, 95% CI 0.16–4.2 6.8% vs 9.1% Adjusted HR 1.89, 95% CI 0.90–3.9	Secondary end point analysis included those with new onset AF after surgery (*n* = 220)
Meurin *et al.* (2008), Int J Cardiol, France [10] Prospective cohort study (level 2b)	Study period: 2002–2005 Follow up: 44 ± 6 days Subgroup analysis (patients in sinus rhythm and without concomitant surgery) Sample: *n* = 185 VKA (target INR 2.0–3.0): *n* = 112 ASA (75–360 mg/day): *n* = 55 No AT: *n* = 18	Subgroup analysis incidence of thromboembolism	No AT (22%) versus VKA (3.5%) versus ASA (0%) No AT versus VKA *P* < 0.01 No AT versus ASA *P* < 0.01	Aim of the study was to identify high risk population in which antithrombotic therapy is needed within the first 6 postoperative weeks Does not analyse data between intervention groups. Only states significance of results between those not receiving antithrombotic therapy and those who received therapy (i.e. VKA, VKA + ASA, ASA)

ACC: American College of Cardiology; ACCP: American College of Chest Physicians; AF: atrial fibrillation; AHA: American Heart Association; APLT: antiplatelet; ASA: aspirin; AT: antithrombotic therapy; CI: confidence interval; EACTS: European Association for Cardio-Thoracic Surgery; ESC: European Society of Cardiology; INR: international normalized ratio; VKA: vitamin K antagonist.

## RESULTS

This best evidence topic acts as an update to that by Asopa *et al.* [2] and looks to provide a renewal of the best evidence following its publication. The authors concluded that 3 months of anticoagulation should remain the standard of care following MVRep, due to the paucity of high-quality evidence supporting the safety of omitting warfarin, together with high rates of AF following discharge [2].

Current guidelines within the area provide mixed recommendations, typically based upon weak evidence or expert consensus. The 2012 American College of Chest Physicians Guidelines recommend antiplatelet therapy for the first 3 months over a VKA, in those patients undergoing MVRep with a prosthetic ring and in normal sinus rhythm [3]. Conversely, the 2021 European Society of Cardiology/European Association for Cardio-Thoracic Surgery (EACTS) make a class IIa recommendation that oral anticoagulation should be considered for the first 3 months after MVRep, regardless of cardiac rhythm, based upon level C evidence [4]. Despite the comparable risk of thromboembolism with aspirin and VKA following MVRep, the high incidence of new-onset AF and resistance to aspirin makes VKAs the preferable option, in view of the lack of randomized data [4]. This recommendation is supported by the previous 2017 EACTS guideline on ‘perioperative medication in adult cardiac surgery’ [5]. The preceding 2008 EACTS recommendations by Dunning *et al.* concluded that patients with indications for anticoagulation (e.g. AF) should be anticoagulated, whereas those without such risk factors may benefit from anticoagulation or antiplatelet therapy alone [6]. Similarly, the 2014 guidance published by American Heart Association/American College of Cardiology gave a class IIa recommendation for the utilization of a VKA in the first 3 months following MVRep, or lifelong in those with risk factors (e.g. AF, previous thromboembolism or hypercoagulable condition) to achieve an international normalized ratio (INR) of 2.5 (range 2.0–3.0), also based upon level C evidence [7]. The more recent 2020 guideline from the American College of Cardiology/American Heart Association provides no formal update to the aforementioned; stating that the beneficial effects seen with antiplatelet agents in bioprosthetic aortic valves may apply to mitral valves, in the context of MVRep. It should be noted that many of the aforementioned guidelines base their recommendations on non-randomized observational studies for aortic bioprosthesis, and not from the literature following MVRep.

A study of significant relevance is that from Paparella *et al.* [8], who performed a retrospective cohort study (*n* = 1882), comparing incidences of arterial thromboembolic events and major bleeding within 6 months following MVRep. The propensity-matched cohort (*n* = 1144) analysed patients treated with VKA (*n* = 858) (data on target INR not reported) and antiplatelet drugs (100 mg aspirin daily) (*n* = 286), excluding those with ongoing or past AF. All patients underwent MVRep with mitral ring implantation; however, patients in the VKA group received more closed ring repairs (67.9% vs 58.3%, *P* < 0.001) and less chordal implantation (9.8% vs 24.5%, *P* < 0.001). Propensity-matched analysis showed significantly higher mortality in those treated with VKA (2.7% vs 0.3%, *P* = 0.02), age [odds ratio (OR) 1.09, 95% confidence interval (CI) 1.05–1.14, *P* < 0.01] and creatinine (OR 6.4, 95% CI 2.2–15.7, *P* < 0.01). Mortality was predominantly due to the association of VKA with major bleeding complications (3.9% vs 0.7%, *P* = 0.01). Multivariate analyses reported associations between major bleeding and female gender (OR 2.5, 95% CI 1.2–5.1, *P* = 0.01) and cross-clamp time (OR 1.02, 95% CI 1.01–1.03, *P* = 0.04). Overall, the authors concluded that anticoagulation was not superior to antiplatelet therapy in preventing thromboembolic complications after MVRep (1.6% vs 2.1%, *P* = 0.50).

Similarly, van der Wall *et al.* [9] in a multicentre, retrospective observational cohort study (*n* = 469) evaluated thromboembolic and bleeding events between patients receiving VKA (target INR 2.0–3.0) (*n* = 325) or aspirin (*n* = 144) (80 mg once daily) in the 3 months following MVRep. They excluded those undergoing concomitant cardiac surgery, or with preoperative AF. Cases of new-onset postoperative AF for >24 h were commenced on low molecular weight heparin and bridged onto VKA if originally receiving aspirin. MVRep involved the implantation of an annuloplasty ring and a variety of concomitant techniques (e.g. leaflet resections, artificial chords tendinae implant, chordal transposition or edge-to-edge technique)—the authors do not provide further details, nor adjusted/sub-analysis. Composite incidences combining thromboembolic and bleeding events were compared as a primary end point. In those without new-onset AF, the cumulative incidence of the primary end point occurred in 8.2% and 8.1% of those receiving VKA and aspirin, respectively (adjusted hazard ratio (HR) 0.97, 95% CI 0.32–2.9). When individually compared, thromboembolism [2.6% (VKA) vs 1.6% (aspirin), HR 0.82, 95% CI 0.16–4.2] and bleeding [6.8% (VKA) vs 9.1 (aspirin), HR 1.89, 95% CI 0.90–3.9] demonstrated no significant difference in incidences between the groups. Importantly, this secondary end point analysis did not adjust for new-onset postoperative AF (*n* = 220, (47%)).

Meurin *et al.* [10], in a prospective, non-randomized, multicentre study of 350 patients undergoing MVRep, looked to report the incidence of thromboembolic complications between groups receiving either VKA (target INR 2.0–3.0), aspirin (75–360 mg/day), VKA and aspirin, or no antithrombotic therapy. Their primary aim was to report the incidence of thromboembolic events in the early period (up to 6 weeks) after MVRep. Repair type was reported by the authors according to the Carpentier classification, with the prosthetic ring being used in all patients. Multivariate was not performed as factors such as Carpentier class, left-ventricular ejection fraction, age and permanent AF were not predicative of thromboembolism. Subgroup analysis was performed to report incidences in patients who remained in sinus rhythm, and without concomitant surgery (*n* = 185). They reported significantly increased rates of thromboembolic events in those not receiving any form of antithrombotic therapy (22%, *n* = 4) when compared to both VKA (3.5%, *n* = 4) and aspirin (0%) individually (*P* < 0.01). Whereas there was no significant difference in the incidence of thromboembolism between the VKA and aspirin groups (*P* = 0.15). Overall, they concluded that the lack of antithrombotic therapy in any form is a predictive factor for thromboembolic events.

The retrospective nature of all the studies within this field, along with the low numbers of patients included, results in numerous limitations and varying degrees of selection bias. Namely, there is a lack of control for surgeon-specific preferences, temporal trends in prescribing practice and adjustment for the type of repair performed. As such, studies and data are lacking in the best practice for those who remain in sinus rhythm following discharge; in addition, the role of novel-oral anticoagulants in those who develop atrial fibrillation remains unexplored.

## CLINICAL BOTTOM LINE

There remains a lack of high-quality randomized studies, controlling for postoperative cardiac rhythm, comparing VKA and antiplatelet therapy in the early postoperative period following isolated MVRep. Current guidelines are based on limited evidence, or expert consensus alone. Based on the currently available evidence, the authors conclude that antiplatelet therapy (e.g. aspirin), is safe and appropriate to use in the 3 months postoperative period following isolated MVRep, in those without preoperative, or postoperative atrial fibrillation. Rates of thromboembolic events are comparable between these patient groups (i.e. VKA versus aspirin), whilst VKA therapy is associated with increased rates of major bleeding events and mortality.

**Conflict of interest:** none declared.

### Reviewer information

Interactive CardioVascular and Thoracic Surgery thanks the anonymous reviewer(s) for their contribution to the peer review process of this article.
